# Non-tuberculous mycobacteria: clinical and laboratory characterisation (2009 and 2019)

**DOI:** 10.1017/S0950268822000899

**Published:** 2022-12-12

**Authors:** Mariana Lopes, Micaela Batista, Teresa Garcia, Helena Alves, Luísa Boaventura, Celeste Pontes, Fernando Rodrigues

**Affiliations:** 1 Department of Infectious Diseases, Centro Hospitalar e Universitário de Coimbra, Coimbra, Portugal; 2 Department of Clinical Pathology, Instituto Português de Oncologia de Coimbra, Coimbra, Portugal; 3 Public Health Unit, Agrupamento de Centros de Saúde Dão Lafões, Viseu, Portugal; 4 Department of Clinical Pathology, Centro Hospitalar e Universitário de Coimbra, Coimbra, Portugal

**Keywords:** Immunosuppression, lung diseases, nontuberculous mycobacteria

## Abstract

A cross-sectional and retrospective study of patients with *Mycobacterium* spp. in a Portuguese tertiary hospital, in 2009 and 2019, was performed to understand better the rise in isolations of nontuberculous mycobacteria (NTM). The number of patients with positive samples for *Mycobacterium* spp. grew from 56 in 2009 to 83 in 2019. The proportion of NTM rose from 39.3% to 49.4% (*P* = 0.240), with *Mycobacterium avium complex* being more frequent in 2009 and *Mycobacterium gordonae* in 2019, and *Mycobacterium tuberculosis complex* decreased from 60.7% to 50.6%. Higher age was associated with NTM in both years, and pulmonary disease and immunosuppression were associated with NTM in 2019 (*P* < 0.05), with weak to moderate correlation (*V* = 0.231–0.343). The overall rise of NTM, allied to their known capacity to resist antimicrobial therapy, alerts clinicians to the importance of recognising potential risk factors for infection and improving future prevention strategies.

## Introduction

The increase in the incidence and mortality from nontuberculous mycobacteria (NTM), registered in the last decades worldwide, represents both a clinical challenge and an emerging public health concern [1–3].

NTM are ubiquitous microorganisms present in soil and water, and human exposure is expected [2, 4]. Although the rising number of isolations may be largely due to better detection techniques and the increasing number of immunosuppressed patients (a risk factor for infection), studies have shown that the number of infections in seemingly immunocompetent patients is rising as well [1]. The diagnosis of pulmonary NTM disease often is established after excluding other causes and based on a combination of clinical and microbiological criteria listed on a clinical practice guideline elaborated by a panel of experts from the American Thoracic Society/European Respiratory Society/European Society of Clinical Microbiology and Infectious Diseases/Infectious Diseases Society of America [5]. Special attention is given to disease severity and extension, microorganism pathogenicity and patient's comorbidities when considering the decision to treat [5].

The public health concern comes with NTM known capacity to resist antimicrobial therapy and its international dispersion [1, 3]. The challenge of the decision to institute therapy and manage said treatment [2], makes it essential to understand better these infections, the patients affected and their evolution.

This study aims to characterise demographic, socioeconomic, clinical and laboratory characteristics of patients with isolation of *Mycobacterium* spp., comparing NTM with those with *Mycobacterium tuberculosis complex* (MTC), and determine the factors associated with NTM isolation, in patients from a Portuguese tertiary hospital, in 2009 and 2019.

## Methods

### Study population and design

A cross-sectional, retrospective, descriptive and analytical study was performed to characterise patients with laboratory isolation of *Mycobacterium* spp. in samples requested in 2009 and 2019 at the *Centro Hospitalar e Universitário de Coimbra (CHUC)*. This Hospital Center has around 1800 beds, provides differentiated care to the central region of Portugal, and includes a transplantation center.

### Data sources/measurements

Clinical data were collected through medical records and laboratory results. The species isolated were considered the dependent variable and grouped into two categories: MTC *vs.* NTM. Each sample is cultured in *Mycobacterium* Growth Indicator Tube, also known as liquid culture, and in Löwenstein-Jensen medium, known as solid culture. The methods used to detect NTM were INNO-LiPA MYCOBACTERIA® in 2009 and GenoType *Mycobacterium* CM® in 2019. To detect MTC was applied AccuProbe® MTC Culture Identification Test in 2009, and LIODetect®TB-ST Tuberculosis Rapid Test and FluoroType® MTB in 2019.

Demo-socioeconomic (age, sex, nationality, socioeconomic status and consumption habits), clinical (pulmonary pathologies, immunosuppression factors, death within six months, treatment decision) and laboratory characteristics (hospital department requesting the sample, type of sample, previous isolation of *Mycobacterium* spp., culture medium, days until isolation and presence of the same species in another sample) were used as independent variables. Low-socioeconomic status was defined by the patient having an income lower than governmental social support. Consumption habits (tobacco, alcohol, intravenous drugs), either active or previous, were obtained in the medical records and classified as a dichotomous variable. Pulmonary pathologies included were asthma, chronic obstructive pulmonary disease, bronchiectasis, interstitial lung disease and cystic fibrosis. Immunosuppression factors considered were chronic kidney disease, diabetes mellitus, chronic hepatic disease, autoimmune disease, active cancer, transplantation, immunosuppressive therapy and human immunodeficiency virus (HIV) infection. Any isolation of *Mycobacterium* spp. reported in the laboratory system in previous years was considered.

### Statistical analysis

Extreme values, measures of central tendency and dispersion were used for numerical variables. Absolute and relative frequencies were used for categorical variables. Chi-square test was applied to assess the associations between categorical variables (or Fisher's test when the assumptions were not met), and t-student test for numerical variables (or Mann–Whitney test when normal distribution was not verified, using the Kolmogorov–Smirnov test with Lilliefors correction). Cramer's V coefficient was executed to quantify the degree of association between categorical variables, and Spearman's correlation coefficient was executed between numerical variables. Binary logistic regression was used to determine the predictive relationship of independent variables and isolated species.

Results were calculated with a 95% confidence interval, and a *P*-value less than 0.05 was considered statistically significant. Statistical analysis was performed using IBM SPSS Statistics 25®.

## Results

### Prevalence of *Mycobacterium* spp. isolates

A list was provided with 79 positive samples for *Mycobacterium* spp. in 2009 and 96 in 2019. Samples not collected in this Hospital Center were excluded. Considering this, the number of patients with isolation of *Mycobacterium* spp. grew from 56 in 2009 to 83 in 2019. MTC was the most isolated species in samples from both 2009 and 2019, despite representing a lower proportion in 2019 (50.6%) when compared to 2009 (60.7%). The rise of NTM proportion from 22 (39.3%) to 41 (49.4%) was not statistically significant (*P* = 0.240). The NTM species identified in 2009 were: *M. avium complex* (10), *M. intracellulare* (3), *M.avium* (2), *M.chelonae* (2), *M.fortuitum* (1), *M.gordonae* (1). In 2019 were: *M. gordonae* (13), *M.intracellulare* (7), *M.avium complex* (3), *M.avium* (2), *M.kansasii* (2), *M. abcessus massiliense* (1), *M.chelonae* (1), *M.chimaera* (1), *M.genavense* (1), *M.simiae* (1). In a total of 12 samples, differentiation of the species was not possible with the method applied (3 in 2009 and 9 in 2019), thus being labelled *Mycobacterium* spp.

Table 1 summarise the characteristics of the study population, divided by year and isolation of MTC and NTM.

**Table 1.** Demo-socioeconomic, clinical and laboratory characteristics in 2009 and in 2019, divided by MTC group and MNT group (*n* = 139)

	2009 (*n* = 56)								2019 (*n* = 83)							
	Total		MTC (*n* = 34)		MNT (*n* = 22)				Total		MTC (*n* = 42)		MNT (*n* = 41)			
	No.	%	No.	%	No.	%	*P*-value	Cramer's V	No.	%	No.	%	No.	%	*P*-value	Cramer's V
Demo-socioeconomical																
Age above 60	23	41.1	10	29.4	13	59.1	**0.027**	0.295	33	39.8	12	28.6	21	51.2	**0.035**	0.231
Sex							0.225								0.326	
*Male*	38	67.9	21	61.8	17	77.3			47	56.6	26	61.9	21	51.2		
*Female*	18	32.1	13	38.2	5	22.7			36	43.4	16	38.1	20	48.8		
Nationality							0.226*								1.000*	
*Portuguese*	49	87.5	28	82.4	21	95.5			76	91.6	38	90.5	38	92.7		
*Other*	7	12.5	6	17.6	1	4.5			7	8.4	4	9.5	3	7.3		
Low-socioeconomic status	16	28.6	10	29.4	6	27.3	0.863		27	32.5	15	35.7	12	29.3	0.531	
Consumption habits	24	42.9	15	44.1	9	40.9	0.813		28	33.7	19	45.2	9	22.0	**0.025**	0.246
*Alcohol*	2	3.6	0	0	2	9.1	0.150*		9	10.8	8	19.0	1	2.4	0.029*	0.267
*Tobacco*	19	33.9	11	32.4	8	36.4	0.757		22	26.5	14	33.3	8	19.5	0.154	
*Intravenous drugs*	6	10.7	5	14.7	1	4.5	0.386*		2	2.4	2	4.8	0	0	0.494*	
Clinical																
Pulmonary disease	11	19.6	4	11.8	7	31.8	0.089*	0.247	27	32.5	7	16.7	20	48.8	**0.002**	0.343
Immunosuppression	34	60.7	23	67.6	11	50.0	0.187		46	55.4	18	42.9	28	68.3	**0.020**	0.256
*Chronic kidney disease*	5	8.9	3	8.8	2	9.1	1.000*		8	9.6	3	7.1	5	12.2	0.483*	
*Diabetes mellitus*	6	10.7	5	14.7	1	4.5	0.386*		9	10.8	6	14.3	3	7.3	0.483*	
*Chronic hepatic disease*	4	7.1	2	5.9	2	9.1	0.642*		3	3.6	3	7.1	0	0	0.241*	
*Autoimmune disease*	5	8.9	4	11.8	1	4.5	0.638*		7	8.4	1	2.4	6	14.6	0.057*	0.220
*Active cancer*	10	17.9	4	11.8	6	27.3	0.167*		17	20.5	5	11.9	12	29.3	0.050	0.215
*Transplantation*	2	3.6	1	2.9	1	4.5	1.000*		3	3.6	2	4.8	1	2.4	1.000*	
*Immunosuppressive therapy*	9	16.1	4	11.8	5	22.7	0.294*		9	10.8	3	7.1	6	14.6	0.313*	
*HIV infection*	15	26.8	9	26.5	6	27.3	0.947		9	10.8	4	9.5	5	12.2	0.738*	
Death	9	16.1	5	14.7	4	18.2	0.727*		14	16.9	6	14.3	8	19.5	0.525	
Treatment	22	39.3	18	52.9	4	18.2	**0.009**	0.348	14	16.9	10	23.8	4	9.8	0.087	0.188
Previous isolation of *Mycobacterium* spp.	11	19.6	9	26.5	2	9.1	0.171*		15	18.1	8	19.0	7	17.1	0.815	
Laboratory																
Requesting department																
*Cardiothoracic surgery*	2	3.6	0	0	2	9.1			0	0	0	0	0	0		
*General surgery*	1	1.8	0	0	1	4.5			1	1.2	1	2.4	0	0		
*Dermatology*	1	1.8	1	2.9	0	0			1	1.2	1	2.4	0	0		
*Infectious diseases*	15	26.8	8	23.5	7	31.8			8	9.6	5	11.9	3	7.3		
*Hematology*	1	1.8	0	0	1	4.5			2	2.4	0	0	2	4.9		
*Allergology*	0	0	0	0	0	0			2	2.4	0	0	2	4.9		
*Intensive medicine*	1	1.8	1	2.9	0	0			0	0	0	0	0	0		
*Internal Medicine*	5	8.9	2	5.9	3	13.6			3	3.6	2	4.8	1	2.4		
*Nephrology*	2	3.6	2	5.9	0	0			1	1.2	0	0	1	2.4		
*Ophthalmology*	0	0	0	0	0	0			1	1.2	0	0	1	2.4		
*Oncology*	0	0	0	0	0	0			1	1.2	0	0	1	2.4		
*Pneumology*	16	28.6	10	29.4	6	27.3			41	49.4	16	38.1	25	61.0		
*Hepatic transplantation*	2	3.6	1	2.9	1	4.5			0	0	0	0	0	0		
*Emergency*	10	17.9	9	26.5	1	4.5			22	26.5	17	40.5	5	12.2		
Sample location																
*Wound aspiration*	1	1.8	1	2.9	0	0			2	2.4	0	0	2	4.9		
*Bronchial aspirate*	14	25.0	8	23.5	6	27.3			28	33.7	14	33.3	14	34.1		
*Biopsy*	3	5.4	2	5.9	1	4.5			3	3.6	2	4.8	1	2.4		
*Sputum*	27	48.2	16	47.1	11	50.0			43	51.8	22	52.4	21	51.2		
*Fragments of tissue*	0	0	0	0	0	0			1	1.2	1	2.4	0	0		
*Blood culture*	2	3.6	1	2.9	1	4.5			0	0	0	0	0	0		
*Bronchoalveolar lavage*	2	3.6	1	2.9	1	4.5			3	3.6	0	0	3	7.3		
*Cerebrospinal fluid*	1	1.8	1	2.9	0	0			0	0	0	0	0	0		
*Pleural effusion*	3	5.4	2	5.9	1	4.5			0	0	0	0	0	0		
*Abscess*	2	3.6	2	5.9	0	0			2	2.4	2	4.8	0	0		
*Gastric juice*	1	1.8	0	0	1	4.5			0	0	0	0	0	0		
*Urine culture*	0	0	0	0	0	0			1	1.2	1	2.4	0	0		
Culture																
*Liquid*	12	21.4	5	14.7	7	31.8			33	39.8	3	7.1	30	73.2		
*Solid*	4	7.1	0	0	4	18.2			3	3.6	0	0	3	7.3		
*Both*	34	60.7	26	76.5	8	36.4			46	55.4	38	90.5	8	19.5		
*Liquid/Contaminated solid*	4	7.1	2	5.9	2	9.1			1	1.2	1	2.4	0	0		
*Liquid/Insufficient for solid*	1	1.8	1	2.9	0	0			0	0	0	0	0	0		
Presence of other samples with *Mycobacterium spp*	25	44.6	21	61.8	4	18.2			20	24.1	16	38.1	4	9.8		

HIV, human immunodeficiency virus; MTC, *Mycobacterium tuberculosis* complex; NTM, non tuberculosis *Mycobacterium*; * Fisher's exact test.

### Sociodemographic characteristics

Median age was higher in 2019 (58.8 years; IQR 47.0–73.0) then in 2009 (55.4 years; IQR 39.5–71.8) and in patients with NTM (62.1 in 2009 and 64.3 in 2019) when compared to patients with MTC (51.0 in 2009 and 52.3 in 2019).

Patients with more than 60 years-old were significantly more in the NTM group, in both years (*P* = 0.027 in 2009; *P* = 0.035 in 2019), although with a weak correlation (*V* = 0.295 in 2009 and *V* = 0.231 in 2019). Male sex predominated in both years, in both groups. Nationality and low-socioeconomic status were not associated with any group of patients. However, it's worth noting that low-socioeconomic status grew in both groups, from 2009 to 2019 (29.4% *vs* 27.3% in 2009; 35.7% *vs* 29.3% in 2019). A total of 24 and 19 patients had consumption habits, in 2009 and 2019, respectively. These were more frequent in patients with MTC in both years (44.1% *vs* 40.9% in 2009; 45.2% *vs* 22.0% in 2019), although the difference was only statistically significant in 2019 (*P* = 0.025), with a weak correlation (*V* = 0.246).

### Clinical characteristics

Pulmonary disease was identified in 32.5% of the patients in 2019 and was more frequent in patients with NTM (31.8% *vs* 11.8% in 2009; 48.8% *vs* 16.7% in 2019). When compared to MTC patients, in 2019, those with NTM were associated with having pulmonary disease (*P* = 0.002) with a moderate correlation (*V* = 0.343).

In 2019, immunosuppression was identified in more patients with NTM (68.3%) when compared to patients with MTC (42.9%), with a significant difference (*P* = 0.020), although with weak correlation (*V* = 0.256). In that same year, autoimmune diseases, including ANCA-associated vasculitis, ulcerative colitis, IgA nephropathy, nephrotic syndrome and rheumatic polymyalgia, were more frequent as well as active cancer, including lung, oesophageal, sigmoid, basal cell carcinoma, BALT lymphoma, Richter syndrome and non-Hodgkin lymphoma of the central nervous system. The opposite was observed in 2009, with immunosuppression being more frequent in patients with MTC (67.6% *vs* 50.0%).

Death within six months of first sample was not significantly different in both years and between groups.

In 2009, 18.2% of patients with NTM received treatment for the infection, and in 2019, only 9.8%. Decision to treat was associated with MTC in 2009 (*P* = 0.009), with moderate association (*V* = 0.348).

A multivariate logistic regression of variables found to be associated with isolation of MNT in 2019 was calculated with a 95% confidence interval (*P* < 0.001; predicting 73.5% of the observations). The odds ratio for NTM was 7.017 for pulmonary disease (*P* = 0.002; CI 2.057–23.939), 4.804 for immunosuppression (*P* = 0.006; CI 1.559–14.801) and 0.238 for consumption habits (*P* = 0.015; CI 0.075–0.755). Age above 60 failed to predict isolation of NTM (OR 1.523, *P* = 0.437; 0.528–4.392).

### Laboratory characteristics

The most frequent departments to request a posterior positive analysis for NTM were Pneumology (27.3% and 61.0%, in 2009 and 2019) and Infectious diseases (31.8% and 7.3%, in 2009 and 2019).

Sputum (48.2% and 51.8%) and tracheal aspirate samples (25.0% and 33.7%) were the most common type of samples that were positive for *Mycobacterium* spp., in both years, for both MTC and MNT. MTC was more frequently isolated in both liquid and solid culture, and NTM only in liquid.

Simultaneous isolation of the mycobacteria in other samples in the same patient was more common in the MTC group. Previous isolation of a *Mycobacterium* spp. was not associated with any group of patients, although being more frequent in MTC patients in both years.

Time between collecting a sample and the isolation of a specie was higher in NTM group than MTC group, specifically 26.14 days *vs.* 12.44 days in 2009 (*P* = 0.001, Spearman = 0.436), and 21.73 days *vs.* 10.62 days in 2019 (*P* = 0.000, Spearman = 0.495).

## Discussion

The gap of a decade allowed the recognition of some differences in the *Mycobacterium* spp. epidemiology. An increase in NTM isolations from 2009 to 2019 was reckoned but without statistical significance. Improvement of laboratory methods to identify these species was significant, and it plays a vital role in this rising tendency [6]. In this study, changing the test from a probe assay to a PCR-fluorescence probe allowed the identification of 4 more species/subspecies in 2019 (10 *vs* 6). Two species potentially pathogenic were newly listed (*Mycobacterium kansasii* and *Mycobacterium abcessus massiliense*), and the total number and proportion of *Mycobacterium avium complex* decreased. The number of NTM isolates with clinical relevance remained the same, despite the difference in risk factors observed, as did the number of cases treated.

The presence of structural lung defects and comorbidities that induce immunosuppression favours colonisation and infection by NTM [7]. These conditions are common in older age patients, where immunosenescence plays an important role. Often diagnosis goes unnoticed and the risk of toxicity related to treatment is higher. A relationship between age above 60 years and NTM isolation was observed in both years. Lung pathology and immunosuppression were associated with NTM isolation in 2019. NTM capacity to develop a biofilm and to resist antimicrobials imposes a higher risk for infection in susceptible patients [7].

The pulmonology department in 2019 provided 61.0% of NTM samples and is responsible for an increase of 20.8% of overall *Mycobacterium* spp. It may be due to the higher diagnostic yield of bronchial aspirate [8]. While for sputum, two positive samples are required for diagnosis, only one is needed in a bronchial wash specimen [5].

Liquid culture media was revealed to be more sensible for isolation of NTM, whereas solid culture provides other information probably identifiable of the mycobacteria. In the presence of a positive Ziehl-Neelsen stain but negative rapid test for tuberculosis, suspicion for isolation of NTM should be raised [4].

Due to its ubiquitous nature and subsequent risk of contamination by NTM, attention to epidemiological context is also required, including profession, recreational activities, addictions and home conditions. For instance, certain consumption habits, such as alcohol and smoking, are known risk factors for infectious diseases, including MTC or NTM [7]. Due to the retrospective nature of this study, it was difficult to evaluate their use clearly in 2009 – however, in 2019, it was possible to establish an association with MTC only. There is a need to better inquire patients about exposure to dust or water recreational environments, since this may help the diagnosis and decision to treat [9].

From a clinical perspective, particularly in pulmonology and oncology areas, doctors' awareness of unspecific symptoms such as cough, fatigue and dyspnoea is essential to screen for NTM colonisation. Given its importance, it is critical to guarantee a better follow-up of patients with lung disease and immunosuppression. A request for high-resolution computed tomography of the lungs and sputum samples as indicated by the British Thoracic Society guidelines [10] is helpful for NTM infection diagnosis do not to go unrecognised. In terms of public health, monitoring for NTM colonisation in recreational places and health care services may diminish susceptible individuals' exposure, and intervening at a community level is fundamental to lower the impact of modifiable risk factors.

## Limitations

The retrospective nature of this study and the low number of provided samples made it difficult to analyse more clinical and epidemiological data and to have a stronger statistical significance. It was evident in multivariate analysis where confidence intervals were wide. The development of a similar study with a higher number of NTM isolations, collected within a longer period, is needed.

Concerning the clinical outcomes, missing data related to treatment occurred due to the loss of follow-up of some patients after obtaining the specimen for analysis.

## Data Availability

Not applicable.
